# Characteristics and Health Status of Informal Unpaid Caregivers — 44 States, District of Columbia, and Puerto Rico, 2015–2017

**DOI:** 10.15585/mmwr.mm6907a2

**Published:** 2020-02-21

**Authors:** Valerie J. Edwards, Erin D. Bouldin, Christopher A. Taylor, Benjamin S. Olivari, Lisa C. McGuire

**Affiliations:** ^1^Alzheimer’s Disease and Healthy Aging Program, National Center for Chronic Disease Prevention and Health Promotion, CDC; ^2^Department of Health and Exercise Science, Appalachian State University, Boone, North Carolina.

In 2015, an estimated 17.7 million U.S. persons were informal caregivers who provided substantial services through in-home, unpaid assistance to their family members and friends (*1*). Caregiving can have many benefits, such as enhancing the bond between caregiver and recipient, but it can also place an emotional and physical strain on caregivers, leading to higher rates of depression, lower quality of life, and poorer overall health (*2*). As the U.S. population continues to age (*3*), the need for informal caregivers will likely increase. However, little nationally representative information on prevalence of caregivers is available. This study examined demographic characteristics and health status of informal caregivers from 44 states,* the District of Columbia (DC), and Puerto Rico, based on data from the Behavioral Risk Factor Surveillance System (BRFSS) collected during 2015–2017. Overall, approximately one in five adults reported that they had provided care to a family member or friend in the preceding 30 days. Fifty-eight percent of caregivers were women, and a majority were non-Hispanic white, with at least some college education, and married or living with a partner. Across all states, 19.2% of caregivers reported being in fair or poor health, although significant state-to-state variation occurred. Caregivers provide important support to family members, friends, and the health care system and might compromise their own health to provide this support (*1*,*2*). Better understanding of caregivers and the challenges they face could inform implementation of improvements in support systems that could enhance not only the health of the caregiver, but that of the care recipient as well. For example, additional data regarding demographics at the state level might aid in more effective planning and support of caregivers with evidence-based programs and assistance (https://www.cdc.gov/aging/publications/features/caring-for-yourself.html).

BRFSS is a random-digit–dialed landline and cellular telephone survey of noninstitutionalized, civilian U.S. adults aged ≥18 years conducted by all 50 states, DC, and three U.S. territories (*4*). Data collected during each calendar month yields a representative sample for the year. Across all states and territories, the weighted median response rate was 45.9% in 2017,^†^ 47.0% in 2016,^§^ and 47.2% in 2015.^¶^

Over a 3-year period (2015–2017), 44 states, DC, and Puerto Rico administered a nine-question module in BRFSS about caregiving to all adult respondents aged ≥18 years. In states where the caregiving module questions were asked in more than 1 year, only the most recent year was included in the analytic data set. The module begins with a screening question: “During the past 30 days, did you provide regular care or assistance to a friend or family member who has a health problem or disability?” Respondents who answered affirmatively were classified as caregivers, and seven additional questions were asked about the main illness or condition of the care recipient, the duration and intensity of caregiving, the level of care needed, unmet needs of the caregiver, and the relationship of the caregiver to the recipient. The remaining question asked noncaregivers (those who responded “No” to the caregiving screening question) to forecast whether they anticipated becoming a caregiver in the next 2 years (Yes/No). As part of the core BRFSS, participants were asked “Would you say your health is excellent, very good, good, fair, or poor?” Information on demographic characteristics reported for caregivers included sex, race/ethnicity (non-Hispanic white [white], non-Hispanic black [black], Hispanic, and other), age (≤44 years, 45–64 years, and ≥65 years), education (high school or less versus some college or more), employment status (employed full time, part time, or self-employed versus all others), and marital status (married or living with a partner versus all others). In addition, the age-adjusted percentage** of caregivers who reported fair or poor health are presented by state. All analyses were carried out using Complex Samples procedure within SPSS Statistics software (version 24; IBM) to account for the weighted data set and complex sampling design.

During 2015–2017, a total of 441,456 U.S. noninstitutionalized adults aged ≥18 years participated in the BRFSS in the 44 states, DC, and Puerto Rico, where the optional caregiving module was administered, yielding 252,602 completed interviews. Overall, 20.7% of respondents were classified as caregivers (95% confidence interval (CI) = 20.2–21.1) (Figure 1). Among those who were not currently caregivers, 16.7% (95% CI = 16.2–17.1) reported that they expected to become caregivers within the next 2 years. The percentage of caregivers across states varied, from 13.7% in Puerto Rico (95% CI = 12.5–15.0) to 28.2% in Tennessee (95% CI = 26.5–30.0). The four states with the highest prevalences of unpaid caregivers (Alabama, Arkansas, Louisiana, and Tennessee) were southern states with >25% of adults identifying as caregivers. Women accounted for 58.1% (56.9–59.3) of unpaid caregivers in all participating states, ranging from 53.0% in Alaska (95% CI = 45.8–60.0) to 62.6% in Maryland (95% CI = 56.9–67.9) (Table). The racial/ethnic characteristics of unpaid caregivers largely mirrored the racial demographics of the states. For example, the majority of caregivers in all jurisdictions except California, DC, Hawaii, New Mexico, and Puerto Rico were white, whereas in Louisiana, Maryland, and Mississippi, blacks represented ≥30% of caregivers and in DC, 57.2% of caregivers. The highest prevalences of Hispanic caregivers were in California, New Mexico, and Puerto Rico, and the highest percentages of caregivers of other races/ethnicities were in Hawaii. Overall, 44.8% of unpaid caregivers were aged <45 years, 34.4% were aged 45–64 years, and 20.7% were aged ≥65 years. However, age distribution also varied by state. In Utah and DC, 55.9% and 54.0% of caregivers, respectively, were aged <45 years, whereas persons aged ≥65 years accounted for 25.4% of caregivers in Florida and 25.1% in Oregon, the two states with the largest percentages of caregivers in this age group.

**FIGURE 1:**
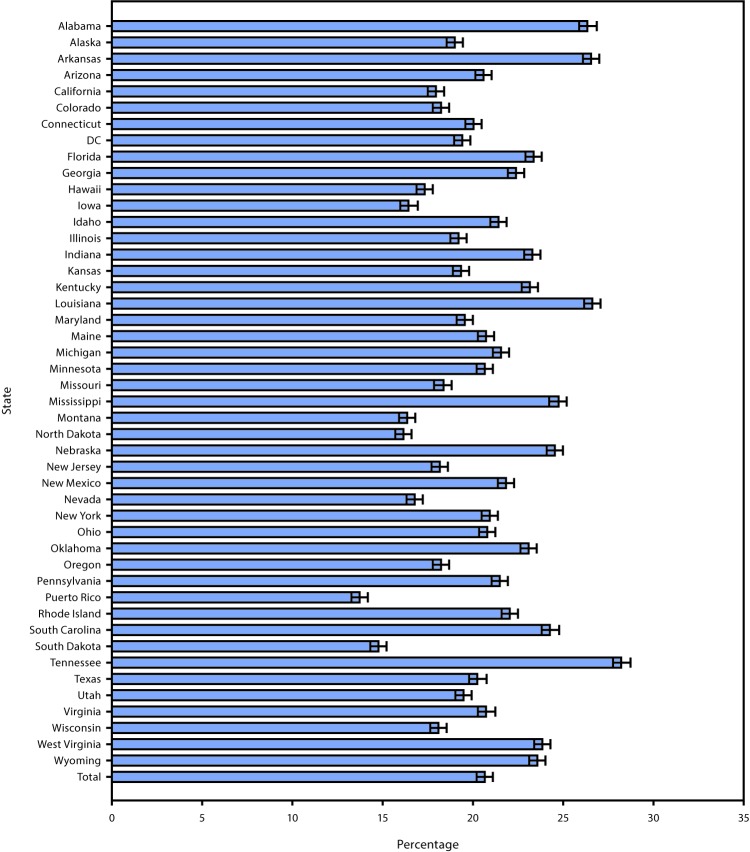
Percentage of respondents self-reporting as informal, unpaid caregivers, by state — Behavioral Risk Factor Surveillance System, United States, 2015–2017 * **Abbreviation:** DC = District of Columbia. * With 95% confidence intervals presented as error bars.

**TABLE:** Demographic characteristics of informal, unpaid caregivers, by state — Behavioral Risk Factor Surveillance System, United States, 2015–2017

State	% (95% CI)											
	Sex	Race/Ethnicity*				Age group (yrs)			Education level		Employment status	Marital status
	Women	White	Black	Hispanic	Other	<45	45–64	≥65	High school or less	Some college or more	Full/Part- time employment	Married/Living with partner
Alabama	57.5	70.8	25.6	1.5	2.1	43.8	35.1	21.2	46.5	53.5	50.2	53.9
	(54.2–60.7)	(67.8–73.6)	(22.9–28.6)	(0.8–2.6)	(1.4–3.1)	(42.1–45.5)	(33.6–36.6)	(20.1–22.2)	(43.3–49.8)	(50.2–56.7)	(48.7–51.7)	(50.6–57.1)
Alaska	53.0	64.8	8.6	4.4	22.2	49.1	34.8	16.1	40.9	59.1	64.3	63.4
	(45.8–60.0)	(56.9–71.9)	(4.1–17.2)	(2.1–9.1)	(16.5–29.3)	(46.0–52.2)	(32.1–37.6)	(14.5–17.8)	(33.9–48.3)	(51.7–66.1)	(61.5–67.1)	(56.3–70.0)
Arizona	60.1	67.7	4.5	21.6	6.2	46.2	31.8	22.0	34.9	65.1	52.4	54.4
	(54.8–65.1)	(62.0–72.8)	(2.2–9.1)	(17.1–26.9)	(4.3–8.8)	(43.6–48.8)	(29.7–34.0)	(20.6–23.4)	(29.6–40.5)	(59.5–70.4)	(49.9–54.8)	(48.9–59.7)
Arkansas	60.2	76.4	13.8	6.1	3.7	43.7	33.9	22.4	53.0	47.0	50.9	59.9
	(54.9–65.2)	(71.1–81.0)	(10.4–18.0)	(3.4–10.6)	(2.2–6.2)	(41.0–46.4)	(31.7–36.2)	(20.9–23.9)	(47.9–58.1)	(41.9–52.1)	(48.5–53.3)	(54.6–65.0)
California	56.1	48.2	7.0	35.5	9.3	47.4	34.1	18.5	30.5	69.5	57.5	52.1
	(49.0–63.0)	(41.1–55.3)	(4.0–12.0)	(28.8–42.9)	(6.3–13.5)	(44.6–50.2)	(31.5–36.8)	(16.8–20.4)	(24.8–36.9)	(63.1–75.2)	(55.0–60.1)	(45.0–59.0)
Colorado	57.9	71.0	3.4	17.0	8.6	48.5	33.2	18.4	28.5	71.5	63.3	61.4
	(53.2–62.4)	(66.1–75.4)	(1.9–6.0)	(13.5–21.2)	(5.8–12.6)	(46.5–50.5)	(31.2–35.0)	(17.3–19.5)	(24.5–33.0)	(67.0–75.5)	(61.5–65.1)	(56.5–66.0)
Connecticut	56.2	70.8	9.4	14.1	5.6	41.9	36.8	21.4	32.6	67.4	59.3	59.1
	(51.6–60.8)	(66.2–75.0)	(6.7–13.1)	(10.8–18.2)	(3.9–8.1)	(39.7–34.9)	(34.9–38.6)	(20.2–22.6)	(28.2–37.3)	(62.7–71.8)	(57.4–61.3)	(54.5–63.7)
Florida	58.0	59.7	15.6	18.7	5.9	41.1	33.5	25.4	41.1	58.9	51.8	55.7
	(52.9–62.8)	(54.6–64.6)	(12.0–20.1)	(15.0–23.2)	(4.0–8.6)	(38.8–43.5)	(33.5–31.5)	(23.9–26.9)	(36.1–46.3)	(53.7–63.9)	(49.7–53.9)	(50.7–60.6)
Georgia	58.2	59.6	28.8	4.8	6.8	43.5	36.4	20.1	43.0	57.0	55.2	56.3
	(53.9–62.4)	(55.3–63.8)	(25.1–32.9)	(3.1–7.4)	(4.4–10.3)	(41.5–45.6)	(34.5–38.5)	(18.9–21.3)	(38.7–47.3)	(52.7–61.3)	(53.4–56.9)	(51.9–60.5)
Hawaii	57.1	21.5	1.8	12.4	64.3	43.1	32.4	24.4	34.7	65.3	62.9	58.3
	(53.2–61.0)	(18.5–24.9)	(0.6–5.4)	(9.9–15.5)	(60.3–68.1)	(41.5–44.8)	(31.0–33.9)	(23.2–25.8)	(30.9–38.8)	(61.2–69.1)	(61.4–64.4)	(54.3–62.1)
Idaho	55.6	83.6	0.4	10.1	5.9	46.0	33.1	21.0	39.1	60.9	58.5	67.2
	(51.3–59.8)	(79.4–87.0)	(0.1–2.8)	(7.2–14.0)	(4.2–8.3)	(43.9–48.0)	(31.3–34.9)	(19.7–22.3)	(35.0–43.3)	(56.7–65.0)	(56.6–60.3)	(63.0–71.1)
Illinois	57.5	71.3	14.6	9.7	4.5	44.6	35.6	19.8	34.8	65.2	58.1	59.8
	(53.1–61.8)	(67.0–75.2)	(11.5–18.2)	(7.2–13.0)	(2.9–6.7)	(42.7–46.6)	(33.9–37.3)	(18.7–20.9)	(30.8–39.2)	(60.8–69.2)	(56.4–59.8)	(55.5–64.0)
Indiana	60.3	85.9	6.9	4.0	3.3	43.4	35.9	20.7	47.5	52.5	56.2	62.5
	(56.1–64.3)	(82.4–88.8)	(4.8–9.7)	(2.5–6.4)	(2.2–5.0)	(41.4–45.5)	(34.1–37.7)	(19.5–21.9)	(43.2–51.8)	(48.2–56.8)	(54.3–58.0)	(58.0–66.7)
Iowa	57.3	90.9	3.0	2.5	3.6	43.0	34.9	22.1	39.2	60.8	61.0	63.9
	(52.9–61.7)	(87.3–93.5)	(1.8–5.0)	(1.1–5.5)	(2.1–6.2)	(41.2–44.8)	(33.5–36.5)	(21.0–23.3)	(34.9–43.7)	(56.3–65.1)	(59.4–62.6)	(59.2–68.3)
Kansas	59.7	80.8	5.0	8.6	5.6	45.5	33.0	21.4	34.2	65.8	62.4	66.6
	(56.8–62.5)	(77.9–83.4)	(3.9–6.4)	(6.5–11.3)	(4.4–7.2)	(44.3–46.8)	(31.9–34.2)	(20.6–22.3)	(31.4–37.1)	(62.9–68.6)	(61.3–63.5)	(63.8–69.2)
Kentucky	56.7	87.2	7.7	1.8	3.4	43.8	35.8	20.4	49.0	51.0	51.6	59.3
	(52.7–60.6)	(83.7–90.0)	(5.5–10.5)	(0.9–3.6)	(1.9–5.9)	(41.9–45.7)	(34.1–37.5)	(19.2–21.6)	(45.1–52.9)	(47.1–54.9)	(49.9–53.3)	(55.3–63.2)
Louisiana	56.1	62.5	30.0	3.3	4.2	45.5	34.6	19.8	49.5	50.5	55.1	51.9
	(51.9–60.1)	(58.3–66.4)	(26.3–34.1)	(2.0–5.4)	(2.9–6.0)	(43.5–47.6)	(32.8–36.5)	(18.6–21.1)	(45.4–53.5)	(46.5–54.6)	(53.2–56.9)	(47.9–56.0)
Maine	56.1	94.4	1.0	1.7	2.8	38.0	38.1	23.9	42.9	57.1	57.4	64.4
	(52.1–60.1)	(91.7–96.2)	(0.3–3.4)	(0.8–3.8)	(1.8–4.6)	(36.0–40.0)	(36.3–22.7)	(22.7–5.3)	(38.9–47.0)	(53.0–61.1)	(55.6–59.2)	(60.4–68.3)
Maryland	62.6	58.8	30.5	3.7	7.0	43.7	35.3	21.0	33.5	66.5	61.8	58.7
	(56.9–67.9)	(52.7–64.6)	(25.2–36.3)	(2.1–6.3)	(3.6–13.1)	(41.0–46.5)	(33.1–37.6)	(19.4–22.5)	(27.8–39.6)	(60.4–72.2)	(59.4–64.1)	(52.6–64.4)
Michigan	54.4	73.0	17.1	2.9	7.0	42.3	35.5	22.2	41.0	59.0	53.5	57.4
	(49.6–59.2)	(68.2–77.3)	(13.4–21.7)	(1.5–5.4)	(4.9–10.0)	(40.1–44.5)	(33.5–37.6)	(20.8–23.7)	(36.3–45.9)	(54.1–63.7)	(51.4–55.6)	(52.5–62.1)
Minnesota	61.0	86.7	5.5	2.3	5.5	44.2	35.2	20.6	29.8	70.2	64.2	65.2
	(59.0–63.1)	(84.9–88.3)	(4.4–6.9)	(1.8–3.0)	(4.5–6.7)	(43.3–45.2)	(34.3–36.1)	(20.0–21.2)	(27.8–31.9)	(68.1–72.2)	(63.4–65.1)	(63.1–67.3)
Mississippi	55.6	58.7	38.8	1.8	0.7	45.9	34.0	20.1	43.9	56.1	49.4	54.0
	(51.3–59.8)	(54.4–62.8)	(34.8–43.0)	(0.7–4.3)	(0.3–1.6)	(43.9–47.9)	(32.3–35.8)	(19.0–21.2)	(39.8–48.2)	(51.8–60.2)	(47.5–51.2)	(49.8–58.1)
Missouri	57.7	83.3	9.8	3.4	3.6	41.8	35.2	23.1	45.0	55.0	58.4	59.6
	(53.2–62.0)	(79.8–86.3)	(7.5–12.7)	(2.0–5.8)	(2.5–5.0)	(39.8–43.8)	(33.4–36.9)	(21.8–24.3)	(40.5–49.6)	(50.4–59.5)	(56.7–60.1)	(55.0–64.1)
Montana	58.2	84.0	0.0	3.9	12.1	41.5	34.6	23.9	37.6	62.4	58.8	62.5
	(53.5–62.8)	(80.1–87.2)	(0.0)	(2.3–6.6)	(9.3–15.5)	39.6–43.5)	(32.9–36.3)	(22.6–25.2)	(33.0–42.3)	(57.7–67.0)	(57.0–60.5)	(57.7–67.0)
Nebraska	58.4	85.4	5.6	4.6	4.5	46.2	33.4	20.4	37.1	62.9	64.7	61.3
	(54.8–62.0)	(81.8–88.3)	(3.7–8.5)	(3.1–6.6)	(2.8–7.0)	(44.4–47.9)	(31.9–35.0)	(19.4–21.5)	(33.6–40.7)	(59.3–66.4)	(63.1–66.2)	(57.7–64.9)
Nevada	54.7	57.5	13.9	13.3	15.3	46.2	33.5	20.3	38.0	62.0	57.7	50.5
	(49.2–60.1)	(51.8–63.0)	(10.1–18.9)	(10.2–17.2)	(10.8–21.3)	(43.9–48.5)	(31.4–35.7)	(18.8–21.9)	(32.7–43.7)	(56.3–67.3)	(55.6–59.8)	(45.2–55.8)
New Jersey	58.8	69.7	12.1	10.0	8.2	43.1	35.8	21.0	36.9	63.1	57.7	61.0
	(53.9–63.6)	(65.2–73.9)	(9.3–15.6)	(7.3–13.5)	(5.9–11.4)	(40.7–45.5)	(33.7–38.0)	(19.7–22.6)	(32.3–41.8)	(58.2–67.7)	(55.5–59.8)	(56.4–65.5)
New Mexico	58.5	42.2	2.0	44.8	11.1	43.9	33.2	22.9	36.5	63.5	51.2	58.5
	(54.4–62.5)	(38.3–46.1)	(1.1–3.5)	(40.7–49.0)	(8.9–13.7)	(42.0–45.9)	(31.5–34.9)	(21.6–24.2)	(32.5–40.7)	(59.3–67.5)	(49.4–52.9)	(54.3–62.5)
New York	60.2	64.5	15.3	12.8	7.4	44.4	34.8	20.7	33.6	66.4	56.4	57.2
	(56.2–64.1)	(60.6–68.1)	(12.5–18.7)	(10.5–15.6)	(5.4–10.1)	(42.5–46.3)	(33.1–36.6)	(19.5–22.0)	(29.7–37.8)	(62.2–70.3)	(54.6–58.1)	(53.1–61.2)
North Dakota	59.7	85.0	2.4	1.6	11.0	47.8	31.8	20.3	36.4	63.6	64.8	63.3
	(55.3–63.9)	(80.8–88.3)	(0.9–5.8)	(0.9–3.1)	(8.3–14.6)	(46.1–49.6)	(30.4–33.3)	(19.3–21.4)	(32.1–40.9)	(59.1–67.9)	(63.2–66.4)	(58.7–67.7)
Ohio	60.6	80.5	11.8	2.8	4.9	43.8	34.9	21.3	43.3	56.7	58.0	52.8
	(56.8–64.3)	(76.8–83.7)	(9.2–15.0)	(1.6–4.9)	(3.3–7.2)	(42.0–45.5)	(33.4–36.4)	(20.3–22.4)	(39.5–47.0)	(53.0–60.5)	(56.4–59.6)	(49.1–56.5)
Oklahoma	60.5	72.7	5.3	4.3	17.6	46.2	32.5	21.3	42.9	57.1	54.8	59.5
	(55.5–65.2)	(67.9–77.1)	(3.5–8.0)	(2.7–6.9)	(13.9–22.1)	(43.7–48.7)	(30.4–34.7)	(19.9–22.8)	(38.0–48.0)	(52.0–62.0)	(52.5–57.1)	(54.5–64.4)
Oregon	56.0	82.5	1.3	7.1	9.1	40.8	34.2	25.1	34.5	65.5	56.0	62.0
	(51.8–60.2)	(78.6–85.8)	(0.5–3.3)	(4.9–10.1)	(6.8–12.1)	(38.9–42.6)	(32.5–35.9)	(23.7–26.4)	(30.4–38.8)	(61.2–69.6)	(54.4–57.6)	(57.8–66.1)
Pennsylvania	58.1	81.4	11.1	4.5	3.0	40.0	36.2	23.7	49.5	50.5	58.7	59.3
	(53.8–62.4)	(77.7–84.6)	(8.5–14.3)	(2.8–7.1)	(1.9–4.8)	(38.1–42.0)	(34.5–38.0)	(22.4–25.2)	(45.2–53.8)	(46.2–54.8)	(57.0–60.3)	(55.0–63.5)
Rhode Island	58.8	81.5	4.4	9.2	5.0	42.3	35.3	22.4	35.2	64.8	57.1	56.7
	(54.4–63.0)	(77.6–84.8)	(2.8–7.0)	(6.8–12.3)	(3.3–7.4)	(40.2–44.4)	(33.5–37.1)	(21.2–23.7)	(31.0–39.7)	(60.3–69.0)	(55.2–59.0)	(52.4–60.9)
South Carolina	57.8	67.3	26.9	2.7	3.1	42.6	34.7	22.6	44.7	55.3	55.6	55.7
	(54.8–60.7)	(64.6–70.0)	(24.4–29.5)	(1.7–4.3)	(2.3–4.1)	(41.2–44.0)	(33.5–36.0)	(21.8–23.5)	(41.8–47.6)	(52.4–58.2)	(54.4–56.9)	(52.8–58.6)
South Dakota	59.5	77.9	1.0	4.8	16.3	43.9	33.8	22.3	44.6	55.4	66.1	63.3
	(53.0–65.7)	(71.0–83.6)	(0.2–5.9)	(1.9–11.4)	(11.7–22.3)	(41.6–46.3)	(31.7–35.9)	(20.7–23.9)	(38.3–51.1)	(48.9–61.7)	(64.1–68.1)	(56.8–69.4)
Tennessee	58.3	75.9	17.5	2.6	4.0	42.5	35.0	22.6	47.1	52.9	56.1	59.4
	(54.6–61.9)	(72.4–79.2)	(14.7–20.8)	(1.3–5.0)	(2.8–5.6)	(40.5–44.5)	(33.2–36.8)	(21.3–23.9)	(43.5–50.7)	(49.3–56.5)	(54.3–57.8)	(55.7–63.0)
Texas	58.7	57.6	10.9	26.7	4.8	51.2	31.9	16.8	36.4	63.6	57.5	59.4
	(52.5–64.7)	(50.9–64.0)	(7.0–16.6)	(21.4–32.7)	(2.5–9.1)	(48.4–54.0)	(29.4–34.6)	(15.3–18.4)	(30.6–42.7)	(57.3–69.4)	(54.8–60.2)	(53.0–65.5)
Utah	62.2	85.4	0.5	9.4	4.6	55.9	28.3	15.8	31.0	69.0	64.8	64.4
	(58.2–66.0)	(82.1–88.2)	(0.2–1.6)	(7.2–12.3)	(3.0–6.8)	(54.2–57.7)	(26.8–29.9)	(14.7–16.8)	(27.2–35.0)	(65.0–72.8)	(63.1–66.4)	(60.3–68.4)
Virginia	57.8	69.6	19.4	5.9	5.1	43.9	36.4	19.7	41.2	58.8	62.7	59.2
	(54.4–61.1)	(66.4–72.7)	(17.0–22.1)	(4.1–8.3)	(3.6–7.1)	(42.4–45.5)	(34.9–37.8)	(18.7–20.8)	(38.0–44.5)	(55.5–62.0)	(61.3–64.1)	(55.9–62.4)
West Virginia	57.7	92.4	3.5	0.9	3.2	40.9	35.5	23.6	52.2	47.8	47.3	60.5
	(54.5–60.9)	(90.2–94.2)	(2.4–5.2)	(0.4–1.9)	(2.1–4.8)	(39.4–42.4)	(34.1–36.9)	(22.5–24.8)	(49.0–55.3)	(44.7–51.0)	(45.8–48.8)	(57.3–63.7)
Wisconsin	55.1	87.5	7.0	3.4	2.1	41.5	36.9	21.6	39.7	60.3	61.9	62.9
	(50.6–59.5)	(83.5–90.6)	(4.5–10.7)	(1.9–6.0)	(1.5–3.1)	(39.5–43.5)	(35.1–38.7)	(20.4–23.0)	(35.5–44.1)	(55.9–64.5)	(60.1–63.5)	(58.4–67.2)
Wyoming	55.2	84.9	0.6	6.4	8.1	45.3	35.1	19.6	38.7	61.3	61.1	62.4
	(50.5–59.8)	(79.8–88.8)	(0.2–1.5)	(3.9–10.3)	(5.2–12.6)	(42.9–47.6)	(33.1–37.1)	(18.4–20.9)	(34.0–43.6)	(56.4–66.0)	(59.0–63.1)	(57.4–67.1)
District of Columbia	58.7	27.3	57.2	9.3	6.2	54.0	29.7	16.3	30.7	69.3	66.1	33.1
	(53.9–63.4)	(23.1–32.0)	(52.1–62.1)	(5.9–14.3)	(4.1–9.4)	(52.0–56.1)	(28.0–31.4)	(15.1–17.5)	(26.4–35.3)	(64.7–73.6)	(64.2–68.0)	(29.1–37.5)
Puerto Rico	62.1	0.8	0.0	98.9	0.2	46.3	32.8	20.9	42.9	57.1	37.8	57.9
	(57.0–67.0)	(0.3–2.1)	(0.0)	(97.6–99.5)	(0.0–1.1)	(44.5–48.2)	(31.1–34.5)	(19.7–22.1)	(38.1–47.9)	(52.1–61.9)	(36.1–39.6)	(53.1–62.6)
**Total**	**58.1**	**67.2**	**12.9**	**13.8**	**6.2**	**44.8**	**34.4**	**20.7**	**39.0**	**61.0**	**56.8**	**57.6**
	**(56.9–59.3)**	**(65.9–68.4)**	**(12.0–13.7)**	**(12.7–14.9)**	**(5.6–6.8)**	**(44.4–45.4)**	**(33.9–34.9)**	**(20.4–21.1)**	**(37.9–40.2)**	**(59.8–62.1)**	**(56.3–57.3)**	**(56.4–58.8)**

**Abbreviation:** CI = confidence interval.

* Whites, blacks, and others were non-Hispanic; Hispanics could be of any race.

Across the jurisdictions, 61.0% of unpaid caregivers reported having at least some college education; Colorado had the highest proportion of caregivers with at least a college education (71.5%), and Arkansas had the highest proportion of caregivers with a high school diploma or less (53.0%). Overall, 56.8% of unpaid caregivers were employed, ranging from 37.8% in Puerto Rico to 66.1% in both DC and South Dakota. An average of 57.6% of caregivers were married or living with a partner, ranging from 67.2% in Idaho to 33.1% in DC.

After age adjustment, 19.2% of caregivers (95% CI = 18.3–20.1) reported being in fair or poor health, although significant state-level variation occurred (Figure 2). Estimates ranged from 11.7% in Minnesota (95% CI = 10.3–13.3) to 34.4% in Puerto Rico (95% CI = 30.4–38.7). In 19 states (Alabama, Alaska, Arizona, Georgia, Indiana, Kansas, Kentucky, Louisiana, Mississippi, Nevada, New Mexico, Ohio, Oklahoma, Oregon, Rhode Island, Tennessee, Texas, West Virginia, and Wyoming) age-adjusted rates of fair or poor caregiver health were ≥20%.

**FIGURE 2:**
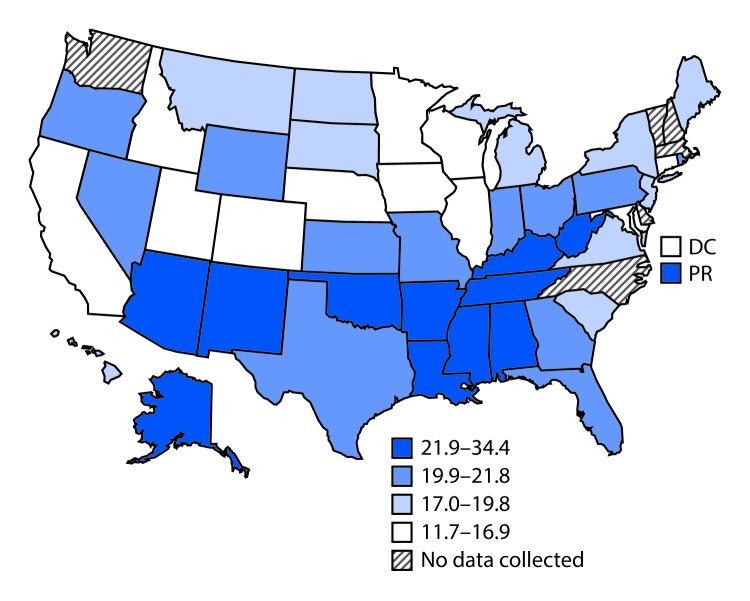
Adjusted percentage* of informal, unpaid caregivers reporting fair or poor health, by state — Behavioral Risk Factor Surveillance System, United States, 2015–2017 **Abbreviations:** DC = District of Columbia; PR = Puerto Rico. *Age-adjusted to the 2000 U.S. Census.

## Discussion

In 44 states, DC, and Puerto Rico, approximately one in five adults reported that they had provided care for a relative or friend in the last month during 2015–2017, suggesting that informal, unpaid caregiving is a widely occurring part of family life in the United States, and that these caregivers play an important role as an adjunct to the formal health care system. In this study, much of the responsibility of caregiving was borne by younger adults (aged <45 years). For younger persons, caregiving might adversely affect their ability to work or negatively affect their income by limiting their work hours or their ability to take on additional job responsibilities (*5*). Among older persons, the physical demands of caregiving might make continued caregiving unsustainable. This study found that the age-adjusted percentage of current caregivers who reported that their health was fair or poor was nearly 20%, with estimates ranging as high as one third in Arkansas and Puerto Rico and >20% in many other states.

As the U.S. population continues to age, the need for informal caregivers is likely to increase. Persons born during 1946–1964 (often referred to as “baby boomers”), who account for a substantial portion of the population, are reaching or are older than age 65 years; in addition, older adults are living longer, with persons aged ≥85 years the most rapidly growing age group (*3*). These circumstances were reflected in the response to the caregiving forecasting question, which found that one in six adults who were not currently engaged in caregiving expected to become caregivers in the next 2 years. Despite the forecasted increase in need for caregivers, population dynamics might result in fewer available caregivers per person for several reasons. First, the number of adult children available per person in need of care is decreasing because of smaller family size (*1*). Second, more adult women, to whom caregiving responsibilities have historically fallen, are currently in the workforce, and therefore might have less ability to become full-time family caregivers. Finally, more families are dispersed geographically, limiting the availability of nearby caregivers (*6*). As these demographic changes are occurring, there is increasing desire among persons born during 1946–1964 to stay in their homes rather than move to senior-oriented housing (*6*); family caregivers likely will be needed to support this option. Recent findings on Alzheimer’s disease have indicated that Alzheimer’s decedents are now more likely to die at home than in institutional settings than they were 15 years ago (*7*); relying on informal caregivers might potentially lower costs to the U.S. health care system (*8*). Given that in many states ≥20% of caregivers describe their current health status as fair or poor, the potential for losing informal caregivers because of poor health exists and needs to be addressed to support caregivers and expanded offerings that allow caregivers to address their own health concerns. The possibility exists that caregivers with fair or poor health might have chosen caregiving because their health has rendered them unable to work in a conventional job. However, given that these data are cross-sectional, understanding this dynamic is beyond the scope of this investigation. Further, the state-to-state variation observed suggests that states and communities might need to tailor efforts to the specific needs of local caregivers.

The findings in this report are subject to at least three limitations. First, information about caregiving was self-reported and might be influenced by social desirability and recall bias. Second, many persons who perform caregiving tasks might not identify their actions as caregiving, but rather think of these responsibilities as part of family living, which could underestimate the number of caregivers. Finally, because BRFSS interviews only one participant per household, a family caregiver who is not the interviewee could be present, thereby undercounting caregivers.

State-specific data might be used to estimate the current scope of caregiving, and for scaling and delivering interventions to support caregivers with state-specific programs. These are the first state-level estimates of self-rated caregiver health. Health care systems could use these data to make organizational updates that account for the important role caregivers have in supporting persons with chronic conditions and disabilities outside health care settings. At the federal level, these findings could inform discussions about ways that caregivers could be supported in federal programs and service delivery Additional data regarding demographics at the state level might aid in planning and supporting caregivers with evidence-based programs and assistance (https://www.cdc.gov/aging/publications/features/caring-for-yourself.html). In all cases, however, these data highlight the need to ensure that caregivers themselves maintain good health; their incapacitation potentially could lead to additional hospitalizations or earlier placement into long-term care of persons who could otherwise be cared for in their home. Proactively addressing the needs of families and caregivers might forestall or eliminate these outcomes. Caregiving can adversely affect the functioning of the caregiver in all domains of well-being (*2*). It can also provide benefits, such as the emotional satisfaction of caring for a loved one, a sense of purpose, financial savings compared with the cost of institutional care, new skills, and increased confidence (*1*,*6*). Caregiving is a public health issue of increasing importance as the U.S. population ages. As public health data systems are modernized, opportunities to analyze data that are more current will expand and should yield more accurate and timely findings to guide policy. Better understanding of caregivers and the challenges they face could inform implementation of improvements in support systems that could enhance not only the health of the caregiver, but that of the care recipient as well.

**Summary**

**What is already known about this topic?**

Informal, unpaid caregivers provide important support to family members, friends, and the health care system and might compromise their own health to provide this support.

**What is added by this report?**

During 2015–2017, approximately 20% of respondents to the Behavioral Risk Factors Surveillance System survey were classified as caregivers. Nearly 20% of caregivers reported fair or poor health, with wide interstate variation, ranging from 11.7% to 34.4%.

**What are the implications for public health practice?**

Because caregiving is a public health issue of increasing importance as the U.S. population ages, the health status of caregivers warrants special attention.

## Footnotes

* Alabama, Alaska, Arizona, Arkansas, California, Colorado, Connecticut, Florida, Georgia, Hawaii, Idaho, Illinois, Indiana, Iowa, Kansas, Kentucky, Louisiana, Maine, Maryland, Michigan, Minnesota, Mississippi, Missouri, Montana, Nebraska, Nevada, New Jersey, New Mexico, New York, North Dakota, Ohio, Oklahoma, Oregon, Pennsylvania, Rhode Island, South Carolina, South Dakota, Tennessee, Texas, Utah, Virginia, West Virginia, Wisconsin, and Wyoming.

† https://www.cdc.gov/brfss/annual_data/2017/pdf/2017-response-rates-table-508.pdf.

§ https://www.cdc.gov/brfss/annual_data/2016/pdf/2016_ResponseRates_Table.pdf.

¶ https://www.cdc.gov/brfss/annual_data/2015/2015_ResponseRates.html.

** Age-adjusted to the 2000 U.S. Census.
